# Readout for simple and precise analog acoustic impact initialization

**DOI:** 10.1038/s41598-021-01090-z

**Published:** 2021-11-08

**Authors:** Roman Sotner, Ladislav Polak, Jan Jerabek, Abhirup Lahiri, Winai Jaikla

**Affiliations:** 1 Faculty of Electrical Engineering and Communication, Brno University of Technology, Brno, Czech Republic; 2 Melexis Technologies, SA, Bevaix, Switzerland; 3 Department of Engineering Education, Faculty of Industrial Education and Technology, King Mongkut’s Institute of Technology Ladkrabang, Bangkog, Thailand

**Keywords:** Electrical and electronic engineering, Acoustics

## Abstract

An economic concept of acoustic shock wave sensing readout system for simple computer processing is introduced in this work. Its application can be found in precise initialization of the stopwatch from the starter sound, handclap or gun in competitive sport races but also in many other places. The proposed device consists of several low-cost commercially available components and it is powered by a 9 V battery. The proposed device reliably reacts on incoming acoustic shock wave by generation of explicit impulse having controllable duration. It significantly overcomes basic implementations using only a microphone and amplifier (generating parasitic burst instead of defined and distinct impulse) or systems allowing a limited number of adjustable features (gain and/or threshold of the comparator—our concept offers the adjustment of gain, cut-off frequency, threshold level and time duration of active state). In comparison with standard methods, the proposed approach simplifies and makes sensing device less expensive and universal for any powder-based starting gun (without necessity to adapt starting gun). The proposed device, among others, has the following features: impulse duration can be controlled from hundreds of μs up to 2.3 s, the gain range of linear part of processing from 6 to 40 dB and open-collector output compatible with 5 V TTL or 3.3 V CMOS logic. The initialization has been tested in the range from tens of centimeters up to four meters. In order to highlight the important spectral components, the spectral character of the signal can be optimally reduced by a low-pass filter. The quiescent power consumption of the designed simple analog circuit reaches 90 mW. Several use cases, response of the designed system on gunshot signature, talking, hand-clapping and hit on the sensing microphone, are studied and compared to each other. Simulation and experimental results confirm functionality of the realized system.

## Introduction

### General discussion

Recently, importance of different techniques proposed to monitor audio event in various areas^1^ is confirmed by numerous works. These works target on sensing of acoustic signal^1,2^, source location estimation^1,2^, acoustic sensor networks^2^ and response analysis including detection of various events^3^. Nowadays, this domain belongs especially to the field of digital signal processing.

In the last decade, significant progress was observed in the signal processing of advanced detection of various types of events included the sensed audio signals, where different kinds of signals have been identified and implemented to specific libraries of the time-domain track samples (see for example^3^) for easy classification of the type of sensed signal. Several interesting works on the topic of gunfire (as a typical representation of strong acoustic impact) localization have been presented in recent years^4–6^, utilizing various methods (independently located sensors in open space (open environment)^4^, a set of microphones receiving direct and reflected acoustic waves in spatial diversity^5^, fulfilling specifications for the field of standard data communication^6^).

Such works confirm that location-specific firing^4,5^ and identification of the time domain (and spectral) patterns^3,6^ are interesting for several research fields. However, not only the detection of impulsive sounds and events, presented in high-quality signals, receives attention^3^. Works presented in^7–10^ are focused directly on the estimation and evaluation of gunfire patterns at various surrounding conditions. Methods presented in these works are able to reveal gunshot pattern in a noisy environment^9^ and under low detectable level conditions^10^. Moreover, some databases of various types of gunshot patterns exist^11^. Such methods exploit digital signal processing and they are quite complex and expensive.

Our work, despite its relation to several above-discussed areas, has different goal. For instance, many sport competitions have a requirement for the precise starting of a stopwatch. An example of use case is competition of non-professional fire fighters teams (started by a gun fire) and natural requirement for a simple and low-cost device. Firefighter’s competition has a specific evaluation of the end of a single race. The stopwatch stops the measurement of time when all “targets” have strike by water flow. It can be detected by a mechanical sensor or general button (it is not a subject of this work and design). Our work targets on the technical improvement of starting initialization. To the best of our knowledge, based on literature survey, no similar device has been reported yet. The proposed sensing device does not require such complicated approaches as presented in^1–6^. Furthermore, our work does not focus on low-intensity fire events mixed with various surrounding noises^3,9,10^. We expect clear evaluation of acoustic shock wave impact at very close position of source and sensor as well as possibility to adjust a proper sensitivity and threshold of activation. An analog-based design ensuring very effective low-cost solution is sufficient in this case as is proved by this research. Works^4,5^, for instance, dealing with localization of the event of gunshot fire, can serve for required purposes after certain modifications. A brief comparison with state of the art with a focus on particular features and also application fields is shown in Table 1.

**Table 1:** Brief comparison of the features of the proposed system and general sound-processing state-of-the-art solutions.

References	Single sensing path (network of sensors not required)	Type of signal processing	Analog to digital conversion not required	Digital processing platform not required	Exact impulse shape identification not required	Purpose (application field)	Expected or tested operational distance	Power consumption	Targeted bandwidth (possible adjustment)	Real time reaction times	Device size	Cost of whole system
^4^	No	Digital	No	No	N/A	Localization	N/A	N/A	N/A	N/A	N/A	High
^5^	Yes	Digital	No	No	No	Localization	Units-tens of meters	N/A	N/A	N/A	Large (computer + tripod + set of microphones)	High
^6^	Yes	Digital	No	No	No	Event detection	Hundreds of meters	N/A	N/A	N/A	Large (Raspberry Pi module + robust desktop microphone	High
^12^	N/A	Analog and digital	No	No	N/A	General sound processing (microphone array)	N/A	N/A	100 Hz–17 kHz (N/A)	N/A	PCB (N/A) incl. analog + FPGA	High
Our work	Yes	Analog	Yes	Yes	Yes	Event initialization	Units of meters	90/216 mW	1.6 Hz–1.6 kHz (Yes)	ms	PCB (70 × 44 mm)	Low

*N/A* not available, *PCB* printed circuit board.

A simple concept of readout (single analog sensing path from several paths of microphone microelectromechanical system based array) for acoustic signals can be found for example in^12^. However, the purpose of the work^12^ is different. Undesired bursts, glitches and overshoots are present in the output waveform produced by an acoustic impact. Application of the concept presented in^12^ for our intentions would lead to ambiguous initialization and detection of an acoustic shock wave. Therefore, a simply sensed and directly amplified waveform from microphone only (without further processing) is inapplicable for our purposes.

The complexity of the analog processing increases with additional blocks for clear initialization of a trigger in comparison to^12^, however, complexity is still very low in comparison to solutions with fully digital processing shown in^4–6^. These digital solutions are applicable for our purposes. However, expected power consumption, size, cost (ratio of effectivity/price) represents the main drawbacks of methods introduced in^4–6^. These solutions are not necessary when simple low-cost analog device provides sufficient features for intended application. Complex digital and mixed-mode platforms represent expensive and unreasonable way when simple low-cost analog solution can be effectively used. These solutions (application for accurate starting of stopwatch) may have issues (in real time operation of digital processing) created by standard low-cost compact microcontrollers. There certain delay is generated by standard operations (sampling, quantization, coding, digital filtering, instantaneous listening/record of data on memory, reading, etc.) as well as by intentional timing for specific software reasons regarding detection of slopes in waveform. Reacting times (from event to generation of resulting output signal for computer) of the presented analog readout are in units of milliseconds.

### Analysis of available solutions of electronic sensing readouts of stopwatch

Manual stopwatch represents the easiest way how to start the measurement of time section. For this purpose, of course, there numerous useful software (SW) applications for different equipment (e.g. smartphones and laptops) exist. However, synchronization of the start of an event with timing of manual pressing of a general button or key on a keyboard is not very good when very accurate time measurement (tens of milliseconds) is required. Typical situation occurs when referee uses a gun for the start of a race and someone else must press a button of electronic stopwatch showing the time on a light board. Some SW applications offer a complete solution including generation of a starting sound^13^. However, these methods are unsuitable for usage with standard powder-based starting guns.

Standard methods for start of electronic timekeeper use a special starting pistol connected to evaluating system directly (by cable), which generates synchronized triggering of a stopwatch^14^. It means that pushing of the gun trigger creates a flash, sets off a bang (acoustic wave generated electronically and propagated by loudspeaker connected to an electronic system), and starts the clock (it also starts the stopwatch)^14,15^. Such concepts have big disadvantage because a specially constructed gun is required. Some previously proposed systems^16^ offer compatibility with any standard gun using powder shells. However, these types require special arrangement for the gun using mechanical pressure sensor to be putted in the pistol barrel^16^.

The full mechanical solution including wireless transmission from the gun to the system significantly increases the expenses of such a concept. The concept containing soundboard and the connected or inbuilt microphone of a computer (laptop) employed as sensors for initialization of a SW-based stopwatch is not the best solution. The SW using external or inbuilt microphone, or external amplifier and Schmidt comparator^17^ may react on undesired signals (with different frequency spectrum, with undesired signal levels or on background noise) and then generates false activation. The sensitivity setting has significant limitation. Such a solution is unreliable when large subsequent changes in a signal (time domain) occur immediately, as it is also discussed in^18^.

On the other hand, there are methods and algorithms to detect, identify and distinguish pattern of sound (caused by a gunshot in many cases), its localization and even classification (of specific type of sound or gun) in acoustic spectrum^19–21^. Unfortunately, such approaches are not operating in real-time as well as the listened and recorded signal must be digitized (digital filtering, sampling, quantization, coding) and then further processed. Therefore, significant delay occurs (unsynchronized measurement) during the signal processing. Moreover, these concepts are unnecessarily robust and have high HW and SW requirements (complex and expensive solution). Consequently, application of these approaches in stopwatches and timekeepers is not very optimal (complex solution of synchronization and requirements on real time response).

Table 2 shows comparison of the methods discussed above in order to show their usability for targeted purpose. Precisely selected systems for localization and source identification purposes are added for visibility of clear differences of these concepts. The analysis of the presented concepts leads to the following conclusions:

- SWs used for time measurement are unsuitable due to unsynchronized manual start of the measurement by pressing of a button after gunfire^13^,
- some special solutions are able to solve the problem completely (a single press of button activating sound and optical effects and initialization of stopwatch simultaneously)^14,15^ but requirements on the gun and whole system construction (it is not universal method allowing to use any type of a starting gun) are high that leads to high costs,
- the sensing device is a wire-connected part of the gun^14–16^,
- only acoustic wave sensing (microphone) principle^18^ offers short-range (reported up to 10 m) wireless application except devices equipped by other radiofrequency communication modules^16^ where the available range is more than several tens of meters,
- the accuracy of common methods reaches units-tens of ms^14–17^,
- many solutions use mixed (analog and digital) design approaches^14–18^ where a dominant part of analog signal processing is also solved digitally^15,16^, which increases demands on the used digital platform and brings additional processing delay and
- power consumptions of the mixed solutions (in majority and if reported) overcomes 1 W and does not expect battery supply (our proposed simple system has more than ten times lower power consumption).

**Table 2:** Comparison of solutions serving for time measurement (evaluation targets on starting conditions) in competitions (comparison also with systems for localization and source identification).

References	Purpose	Solution	Character of the system	Principle	Automatic start	Complexity of the hardware solution	Starting initialization	Immunity on false activation	Synchronization of start of measurement	Real-time operation	Without processing delay of used methods	Sensing device does not require mounting on a gun (source of sound)	Wireless measurement (implemented or possible)	Tested or available distance from the gun to the processing station (computer)	Accuracy (reacting time or delay of the system in our case)	Supposed for low-capacity battery supply	Power consumption
**Sensing readouts in systems for time measurement**																	
^13^	Stopwatch	SW program	–	(a)	No	N/A	Pressing key	N/A	No	Yes	N/A	–	–	–	N/A	–	–
^14^	Stopwatch	HW + SW	Mixed	(b)	Yes	High	Flashing	Yes	Yes	Yes	Yes	No	No	–	Units of ms	No	N/A
^15^	Stopwatch	HW + SW	Mixed	(b)	Yes	High (Arduino platform)	Flashing	Yes	Yes	Yes	Yes	No	No	–	Units of ms	No	N/A
^16^	Stopwatch	HW + SW	Mixed	(c)	Yes	High (PIC microcontrollers + IQRF module + PC), i.e. 100 + components (including chips with high integration density)	Mechanical pressure	Yes	Yes	Yes	Yes	No	Yes*	40 m	Tens of ms	No	> 1 W
^17^	Stopwatch	HW + SW	Mixed	(d)	Yes	High (Atmel platform), i.e. 100 + components (including chips with high integration density)	Acoustic wave	No	Yes	Yes	Yes	N/A	No	N/A	Tens of ms	No**	> 3 W
^18^	Sound initialization	HW + SW	Mixed	(e)	–	High (analog, PIC microcontroller)	Acoustic wave	N/A	Yes	Yes	Yes	Yes	Yes	9 m	N/A	N/A	N/A
**Sensing readouts in systems for localization and source identification purposes**																	
^12^	Sound imaging	HW + SW	Mixed	(f)	–	High (ADC, FPGA)	–	–	–	N/A	N/A	Yes	Yes	13 cm	N/A	No	N/A
^19^	Localization	HW + SW	Mixed	(g)	–	High (ASIC, FPGA)	–	–	–	No	No	Yes	Yes	2 m	N/A	N/A	0.11 W
^20^	Localization	HW + SW	Mixed	(h)	–	High (ARM Cortex platform, robust ADC, GSM transceiver, GPS modules)	–	–	–	No	No	Yes	Yes	10 m	Hundreds of μs***	N/A	N/A
^21^	Identification	HW + SW	Mixed	(i)	–	High (ADC + microprocessor, Raspberry platform)	–	–	–	No	No	Yes	Yes	20 m	N/A	N/A	N/A
Proposed	Stopwatch	HW	Analog	(j)	Yes	Low (few simple analog parts)	Acoustic wave	Yes	Yes	Yes	Yes	Yes	Yes	4 m	4 ms	Yes	0.09–0.22 W

*ASIC* application specific integrated circuit, *FPGA* field programmable gate array.

*Wireless operation ensured by a complex radiocommunication module.

**Discussed as suitable for auto-battery (12 V, > 40 Ah).

***Reported as delay in communication between blocks (no delay in evaluation and synchronization as mentioned in other cases).

(a) SW-based counter (clock)—infinite loop with condition; (b) a special electronic gun initializing timer and creating light and sound effects; (c) a pressure sensor in special arrangement for a standard starting gun; (d) an acoustic microphone and a simple comparator with hysteresis; (e) two stages of amplifiers, peak detector, switches; (f) multichannel sound imaging (the matrix of microphones processed separately); (g) multichannel localization (direction and distance of the source of sound) calculating result from time delays in each channel (operating when SNR = 10 dB, amplifiers + Schmidt comparator in 6–8 channels); (h) multichannel localization including patter detection and distinguishing of the source of sound, i.e. processing of a full record of wave; (i) identification of the source of sound, i.e. processing of a full record of wave; (j) an acoustic microphone and analog processing chain with amplifier, filters, comparator and trigger.

As it is visible, the state-of-the-art solutions requiring full recording of the sensed wave (for localization^19,20^ and identification purposes^21^) do not operate in real time, use complex and robust platforms, their signal processing delay may significantly influence the accuracy of measurement and usually are not targeted on battery supply (low-power). The distance aspects are various and not reflecting the real limits of systems because many works report conditions of the used setup instead of limits of the tested device. Generally, the distance of correct operation falls into a range of units-tens of meters.

### Technical problem identification and motivation

As it was discussed in previous section, many solutions for acoustic wave signal processing (regarding gunshot) target on the detection and localization of the source of event (the source of sound and its localization). These methods require SW-based digital signal processing (pattern detection) and also additional algorithm for evaluation of several signals from several sources (multiple way of sensing and recording)^12,19,20^. These processes represent tasks for computer or robust hardware. Despite usefulness and clear advantages, there are some examples of solutions^15–18^ (using more advanced analog parts than the easiest concepts) targeting to our purpose (timekeeper). However, these approaches are still very complex.

Table 3 contains comparison of the analog parts of the previously discussed systems for stopwatch as well as for localization and identification purposes^12,17–21^. Such a comparison showed that:

- the simplest solution uses a microphone and amplifier that has significant drawback (not improved immunity against false activation^12^)—the system requires further signal processing in the digital part that increases delay,
- simple systems use an electret microphone, amplifier and comparator with hysteresis^17,19^ for minimization of false initialization, however, this modification is still insufficient for fast high changes of the amplitude in the sensed wave,
- peak detectors in combination with amplification and switches are also used^18,20,21^ but still insufficiently because they do not solve repeating of initializing condition after larger time (hundreds of ms) as well as existence of initializing event having lower levels causing long time of integration (inaccuracy),
- only systems presented in^17–19^ generates TTL impulses compatible with digital inputs (other systems serve for linear processing) and
- very low degree of freedom of important parameters (e.g. gain and threshold) simultaneously is available in standardly used concepts^17–19,21^.

**Table 3:** Comparison of analog parts (where applicable) used for acoustic wave processing (by a microphone).

References	Number of analog blocks	Types of the used active elements	Number of the used active elements	Number of available features for adjustments (modification of processing)	Available adjustments (trimmers or value redesign)	Form of the output information	Improved immunity against random initialization	Bandwidth [kHz]	Settable bandwidth (cut-off frequency)	Settable gain	Gain value/range [dB]	SNR evaluating own noise of the linear part [dB]	Principle (blocks in cascade)
^12^	1	OA	2	N/A	N/A	N/A	–	25	N/A	N/A	N/A	59	Electret microphone, 2 amplifiers
^17^	1	OA, BJT	2	1	Threshold level of comparator	TTL impulse	No	N/A	No	No	N/A	N/A	Electret microphone, comparator with hysteresis and switch
^18^	2	BJT	4	1	Sensitivity and gain	TTL impulse	N/A	N/A	N/A	Yes	N/A	N/A	Electret microphone, 2 stages amplifier, peak detector, switches
^19^	3	OA	4	2	Sensitivity and gain, threshold level of comparator	TTL impulse	No	5.5	No	No	N/A	50	Electret microphone, adjustable amplifier, Schmidt comparator
^20^	1	OA	1	N/A	N/A	Impulse	No	N/A	No	No	N/A	N/A	Electret microphone, OA-based peak detector
^21^	4	OA	N/A	2	Sensitivity and gain, cut-off frequency of the filter	N/A	–	16	Yes	No	26	54	Microphone, amplifier, filter, peak detector, RC all-pass delay
Proposed	5	OA, comparative OA, NE555, BJT	5	4	Sensitivity and gain, cut-off frequency of the filter, threshold level of the comparator, time of active state of flip-flop	Generation of long square TTL impulse (adjustable duration)	Yes	1.6	Yes	Yes	6–40	79*	Electret microphone, input limiter, adjustable amplifier, low-pass filter, Schmidt comparator, flip-flop

*BJT* bipolar junction transistor, *OA* operational amplifier, *SNR* signal to noise ratio (noise produced by the system or microphone in silence).

*Estimated from simulation for effective output noise voltage 27 nV/sqrt(Hz) that yields 1.1 uV in a bandwidth of 1.6 kHz and effective output voltage 10 mV: this should be valid without microphone. Otherwise, as in other cases, similar values (valid for microphones) should be considered.

Findings from the above discussion indicate that improvement of the analog part of sensing device in the point of clear initialization and adjustable parameters allows optimal setting of the sensing device. Our solution includes only very simple analog sensing device compatible with the digital input of a computer port (the analog signal is not distributed for its complete digital processing as in^12,19–21^). Furthermore, low power consumption, sufficient distance operationability, sufficient accuracy and reaction time, and wireless operation (mounting of the sensing device on the gun is not necessary as in^14–17^) are among its advantages. Our work significantly improves clear initialization of the input event (e.g. gunshot signature or hand-clapping) by additional flip-flop circuit (as can be seen in the corresponding figures later). It prevents generation of several random initializations because the monostable flip-flop cannot generate any further impulse for very long time (seconds in our case). After that the SWs for stopwatch (some of them are discussed in^13,14^) do not allow further initialization at digital input when time measurement runs. Compared to previous solutions, our proposal offers four adjustable parameters (other approaches have only one or two adjustable parameters), namely: sensitivity and gain, cut-off frequency of the filter, threshold level of the comparator and time of the active state of flip-flop filter (time width of the generated impulse). Note that immediately tunable parameters (trimmers) are gain and time of active state.

Technical issues of commonly used analog methods (in Table 3) can be found in:

- lack of wireless operation (the sensing device must be mounted on the gun or designed as a part of the gun in many cases—not universal and incompatible with any type of a gun),
- low immunity of the device against random initialization or undesired signals,
- high power consumption,
- unclear definition of the generated signal of the initialized state (TTL output),
- unsynchronized initialization with large delay and reacting time from the gun fire and start of time measurement,
- requirements of SW for additional processing causing undesired delay (especially fully listening and recording methods using analog-to-digital converter (ADC) for further processing) and
- lack of additional adjustment of several features improving immunity against other sources of acoustic signal (talking, music, noises, etc.).

Based on the above discussions, design requirements on our concept of sensing readout device are the following:

- real-time operation without significant processing delay,
- clearly defined TTL/CMOS (5/3.3 V) signal for digital input (serial port, USB, etc.),
- no necessity of any SW processing including ADC,
- no necessity to solve time synchronization of the source signal and the receiving system or evaluating SW,
- operationability in a wired placement of the sensing device mounted on the gun or wirelessly (up to several meters—device not mounted on gun),
- short-range wireless operation without radio communication module (additional power consumption) or other transmission of the sensed information (standard microphone should be sufficient),
- low power consumption suitable for low capacity battery supply (9 V),
- possibility to improve low signal to noise ratio (SNR) when undesired signals occur (sensitivity/gain, bandwidth/cut-off frequency, thresholds, time of active state of generated impulse),
- compatibility with any type of powder-based starting gun and
- usage of low-cost commercially available components.

The rest of this paper is organized as follows. The basic concept of the proposed sensing readout system and the use cases of its application are introduced in “Readout circuit for precise acoustic event initialization” section. The signal processing chain of our readout sensing device is explained in “The readout sensing device” section. This section also includes simulation- and measurement-based verification of the designed parts of designed readout system. Three different sources of events for initialization are tested and analyzed in “Experimental verification of different use cases” section. Simulation-based behavior of the system working with a sample of signal having signature of gunfire is presented in “Processing of gunfire signature” section. The paper is concluded in “Conclusion” section.

## Readout circuit for precise acoustic event initialization

**Figure 1:**
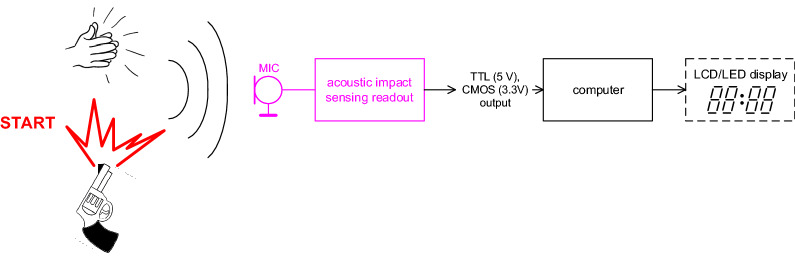
Principle of the sensing readout system for acoustic wave initialization.

Block diagram of the proposed readout circuit for precise acoustic wave initialization is shown in Fig. 1. After a single strong acoustic event, a strong acoustic shock wave is detected by a microphone. The readout part transforms the wave into an electrical signal in the form of single impulse easily adaptable for TTL (5 V) and CMOS (3.3 V) levels. Next, the impulse initializes the digital counter of timer (big LED or LCD segments visible for racers as well as visiting audience) connected to a computer or laptop. We assume that the source of acoustic wave and the detector (microphone or the whole readout device) are close to each other (less than 4 m). Moreover, the device or microphone can be even fixed on the source (e.g. a gun) tightly.

The readout (see the block in purple color in Fig. 1) can be divided onto the following parts: (a) sensor (an electret microphone), (b) limiter of the level of input signal (expectation of various types of signal sources can be connected), (c) amplifier with settable gain (for amplitude sensitivity setting), (d) RC filter (bandwidth limitation to very low frequencies concerning low-frequency character of a shock wave), (e) comparator with large hysteresis (preventing accidental activation for fluctuations in a sensed signal—first protection) and, finally, (f) monostable flip-flop circuit (trigger) performing generation of a long impulse and also preventing random activation (second protection).

## The readout sensing device

**Figure 2:**
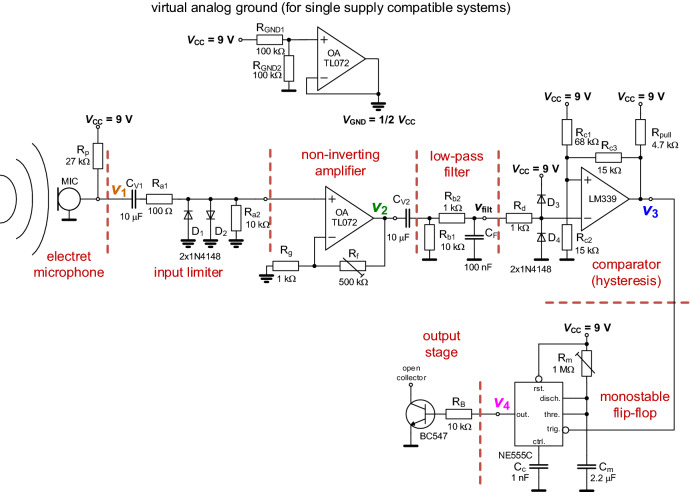
The complete circuit topology of the sensing system designed for initialization of acoustic impact event.

The complete circuit topology of the analog-based sensing system (readout part from Fig. 1) is shown in Fig. 2. It consists of basic building blocks connected in row starting from microphone and ending with output stage. Some of included stages are standard topologies of circuitries, used in different fields of signal processing. All stages are logically interconnected to a system for processing of the signal from microphone. The realized system combines linear as well as intentional nonlinear signal-processing operations.

A standard electret microphone^22^ was selected as the source of signal that is further processed by the readout system. The microphone requires DC bias supply from standard power supply (9 V) through resistor *R*_p_ = 27 kΩ (based on recommendation presented in^22^). The diode-based limiter is complemented by a simple resistor divider (*R*_a1_ and *R*_a2_) and by AC coupling. It represents the first circuitry (stage) after microphone connected to the system. Resistor *R*_a2_ also clearly specifies the input resistance of the amplifier. Hence, the cut-off frequency of AC coupling between stages (high-pass response) will be *f*_l(−3 dB)_ ≅ 1/(2·π·*R*_a2_·*C*_V1_), in our particular case around 1.6 Hz. The diode limiter (approx. ± 0.6 V) is sufficient for expected signal levels from various types of microphone and protects the input of the operational amplifier (OA)^23^. Analog ground created by the second OA in a single package of TL072^24^ as a half of the power supply voltage (4.5 V) is important for circuitry of the noninverting amplifier^24^. This topology of the amplifier was selected due to requirement on gain, which must be always >> 1 due to the low levels coming from microphone (units of mV). Indeed, topology of non-inverting amplifier does not allow lower gains.

**Figure 3:**
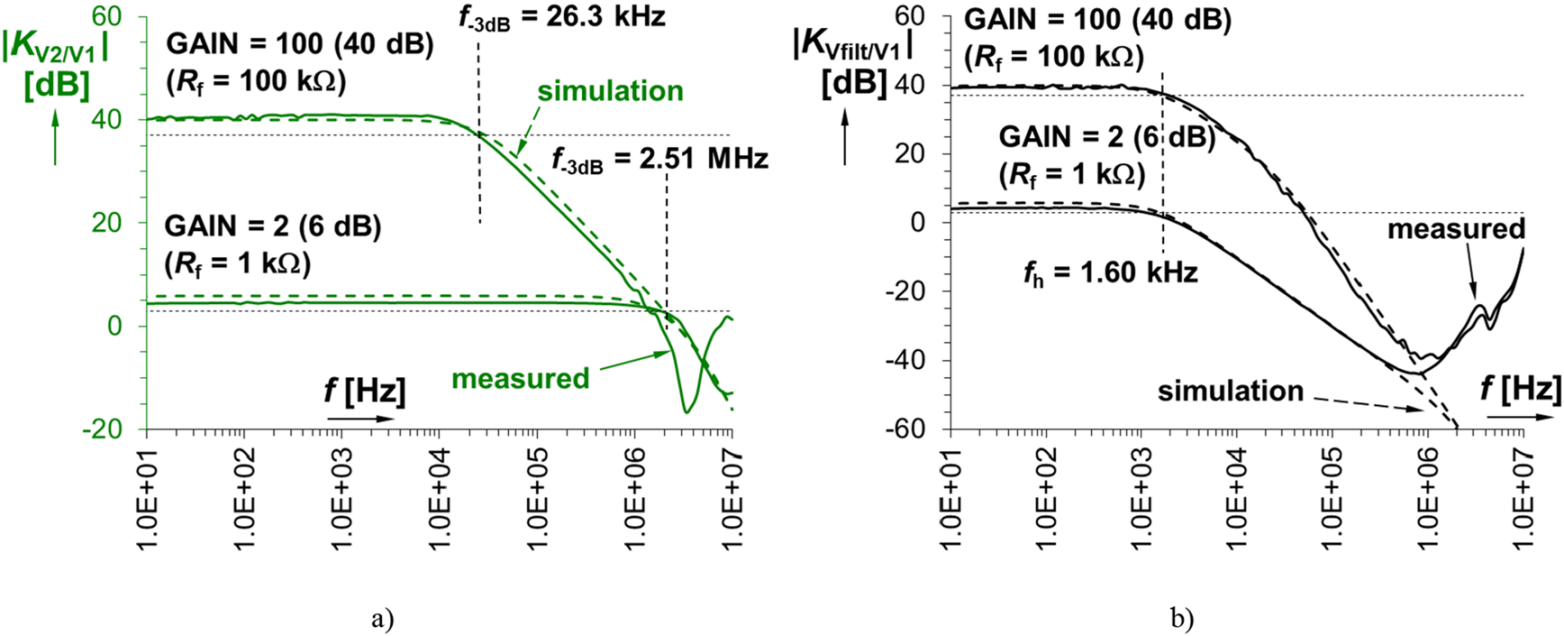
The magnitude frequency response (transfer) of the linear part: (**a**) input amplifier, (**b**) amplifier and filter together.

The OA is followed by a simple passive low-pass first-order filter (excluding coupling C_V2_, *R*_b1_ creating a very low cut-off frequency as discussed above) used for limitation of the spectral character of the output voltage *V*_2_ up to 1.6 kHz (*f*_h(−3 dB)_ ≅ 1/(2·π·*R*_b2_·*C*_F_). The filter, which can be tuned very close to the basic harmonic of the sensed waveform, significantly helps at the interference (decreased SNR) with other strong signals (such a filter is not often presented in recent designs of sensing devices, see Table 3, except^21^). Therefore, the bandwidth limitation provides important improvement of the features of the sensing device. The magnitude frequency responses of the signal processing chain consists of AC coupling, limiter, amplifier and filter are shown in Fig. 3 (left part shows response of the amplifier, right part covers response of the amplifier and filter in cascade together). The filtering and amplifying blocks could be solved also by a single opamp-based structure.

However, we have to ensure high input impedance and independent setting of gain of the amplifier for the same (unchanged) cut-off frequency that would not be available in case of a single opamp-based low-pass filter. The experimental cut-off frequencies of the amplifier reach 26.3 kHz and 2.51 MHz for gain of 40 dB and 6 dB, respectively. The exemplary gains (*K*_V2_ = 1 + *R*_f_/*R*_g_) are set on values 2 (6 dB) and 100 (40 dB), respectively, because the expected range of gains in the processing chain falls into this range. The theoretical value of maximally settable gain reaches almost 54 dB (*R*_f_ = 500 kΩ), but such a large value is not necessary.

The DSOX-3024T oscilloscope was used for recording of the presented results. The selected experimental results are also confirmed by simulations realized in PSpice (OrCad 16.6).

**Figure 4:**
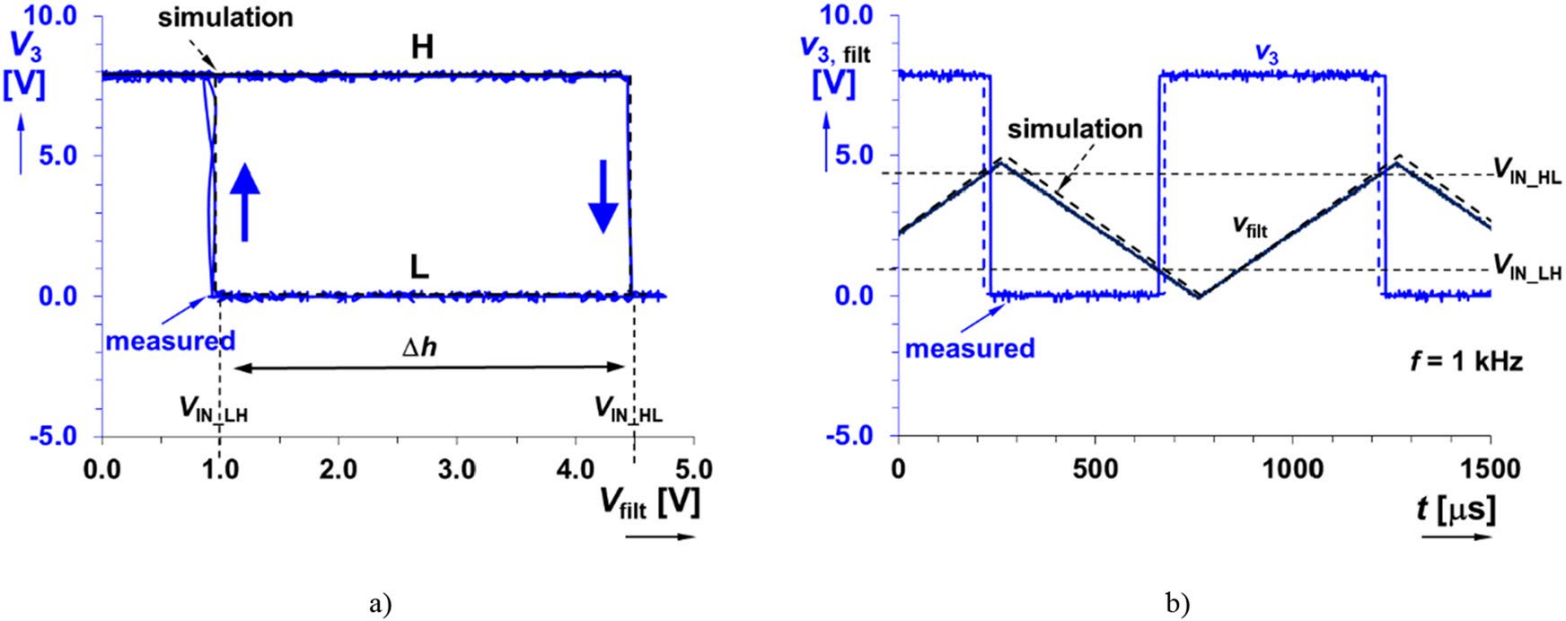
Test of the comparator stage in both simulation and measurement: (**a**) hysteresis diagram, (**b**) time-domain.

The next stage is the comparator with hysteresis. It is a standard concept using the open-collector output of the LM339 device^25^. The values of resistors, considering *R*_c3_ = 15 kΩ (initial selection), are obtained as follows: *R*_c1_ ≅*R*_c3_ · (*V*_IN_HL _− *V*_IN_LH_)/*V*_IN_LH_ and *R*_c2_ ≅*R*_c3_ · (*V*_IN_HL _− *V*_IN_LH_)/(*V*_OUT_H_ − *V*_IN_HL_)^26^. These equations (and real behavior) result in obtaining of *R*_c1_ = 68 kΩ and *R*_c2_ = 15 kΩ for *V*_IN_HL_ = 4.5 V and *V*_IN_LH_ = 1 V respectively, when we expect *V*_OUT_H_ = 8 V (influence of pull up resistor having comparable value to working elements). The pull-up resistor (*R*_pull_) equals to 4.7 kΩ (value recommended by datasheet). The hysteresis loop for this design is depicted in Fig. 4a. The values of threshold voltages, *V*_IN_HL_ = 4.46 V and *V*_IN_LH_ = 0.95 V, were obtained experimentally. This arrangement is necessary in order to prevent unintentional switching under the threshold of initialization (superposed noise created by talking, music, etc. supposed in low hundreds of mV). Figure 4b shows an example of experimentally tested comparator performance by input waveforms at frequency 1 kHz.

**Figure 5:**
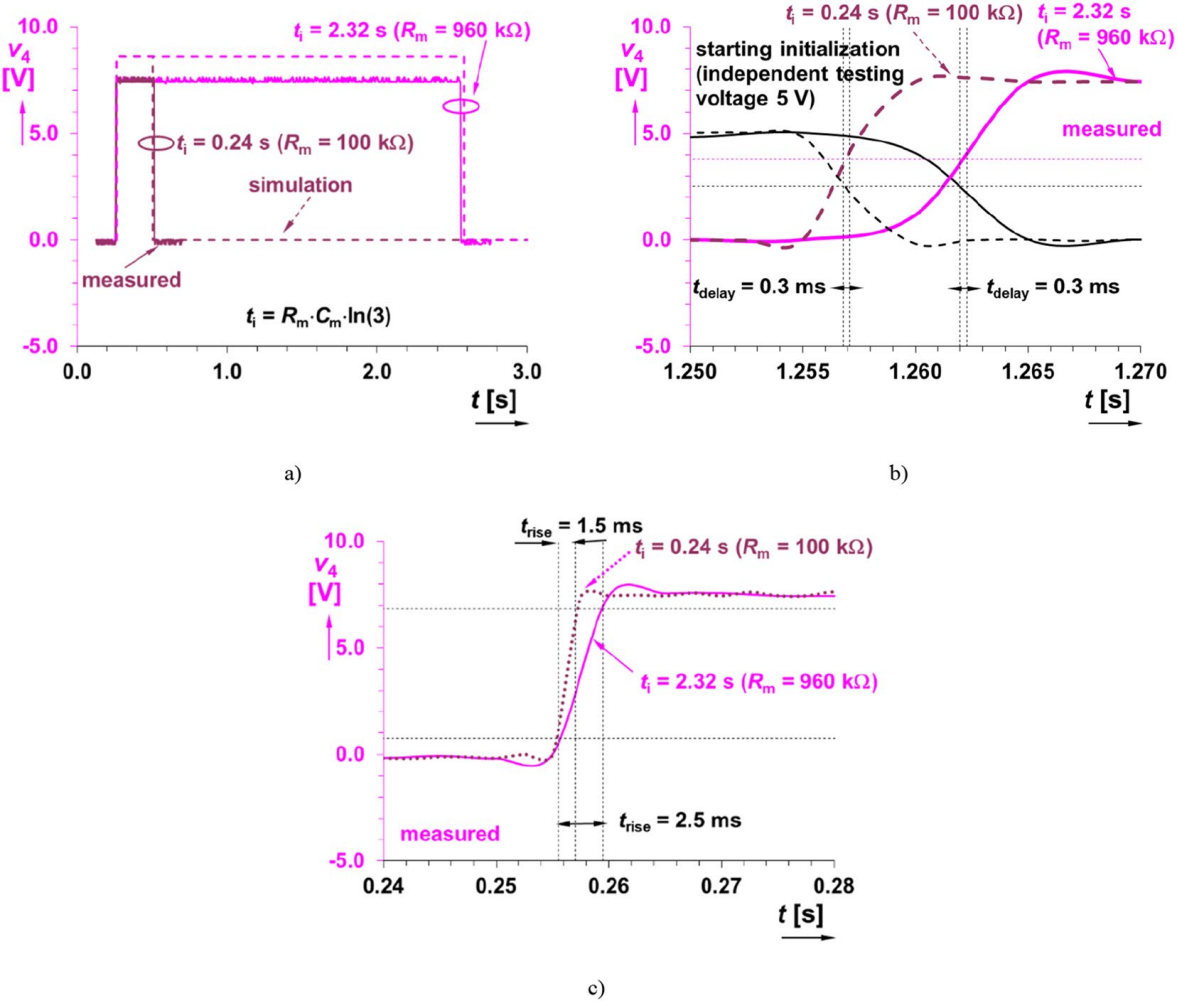
Time domain example of various interval of temporarily stable state of monostable flip-flop: (**a**) full impulse scale, (**b**) detail on initialization for delay and (**c**) detail on initialization for rise time.

The monostable flip-flop circuit employing NE555C^27^ is also used in its standard topology. The flip-flop circuit is required due to specification of duration (adjustable) for active state (shoot initialized) as well as for clear definition of the start of impulse. The sensed shock waveform from the microphone *v*_1_(*t*) has really very variable behavior that generates several impulses (depending on intensity) by the comparator (*v*_3_(*t*)). The amplifier can be even saturated but it is not issue. The interval of temporarily stable state^27^ is defined as *t*_i_ = *R*_m_·*C*_m_·ln(3), which at *C*_m_ = 2.2 μF (selected) can be adjusted by *R*_m_ up to 2.32 s (considering experimentally tested maximal value of the trimmer *R*_m_ = 960 kΩ). The lowest value of *t*_i_ can be approximately set to 300 μs (by *R*_m_ < 100 Ω). This limit depends on the features of NE555^27^. The example of operation in time domain for *R*_m_ = 100 kΩ (*t*_i_ = 0.24 s) and *R*_m_ = 960 kΩ (*t*_i_ = 2.32 s) is shown in Fig. 5 (both cases—two pairs of traces). The units of seconds were selected for clear initialization of the detected event by LED diode lighting during the whole *t*_i_ period. Of course, these properties can be easily modified till limits of the system (NE555C). Slight difference between the simulated and measured amplitudes is caused by the declared variability of the output level of real NE555 (up to 2–3 V is possible as noted in^27^). The output open-collector transistor in the output stage (see Fig. 2) allows connection to the TTL/CMOS compatible input port of further stage by an additional pull-up resistor related to different power supply or H level. The time delay between initializing event and response of the generated impulse reaches approximately 400 μs for trigger block.

Some of the used devices can be replaced by more recent ones having better features in some ways. However, it is not necessary due to minimization of costs, intended operational bandwidth and levels of processed signals.

## Experimental verification of different use cases

The correct setting of sensitivity of the readout allows precise initialization on signal overcoming adjustable detectable threshold. The behavior of the whole developed sensing readout system was tested for three use cases: talking (the distance between the device and microphone is 0.5 m), handclap (up to 0.5 m from the device), and direct hit on the microphone. Distance of 0.5 m sufficiently fulfills requirements on operation because sensing device is supposed to be placed very close to the acoustic source (even the readout will be mechanically connected with the source). The sensitivity of the readout is adjusted (*R*_f_ = 16 kΩ, i.e. the gain is 25 dB in all cases presented here; threshold of reaction on input level larger than approximately 200 mV) for initialization on handclap at distance approximately 0.5 m (and closer).

### Talking

The readout is able to react on talking and other kind of sounds. However, the signal detectable by the system is limited (for all tests) to low frequencies (up to 1.6 kHz as default value). It is partly because of spectral character of tested sounds, talking, clapping, etc. Sensitivity of the device has been set above common sound levels such as voice signals. Moreover, the bandwidth of the low-pass filter can be easily modified for appropriate operation when necessary.

**Figure 6:**
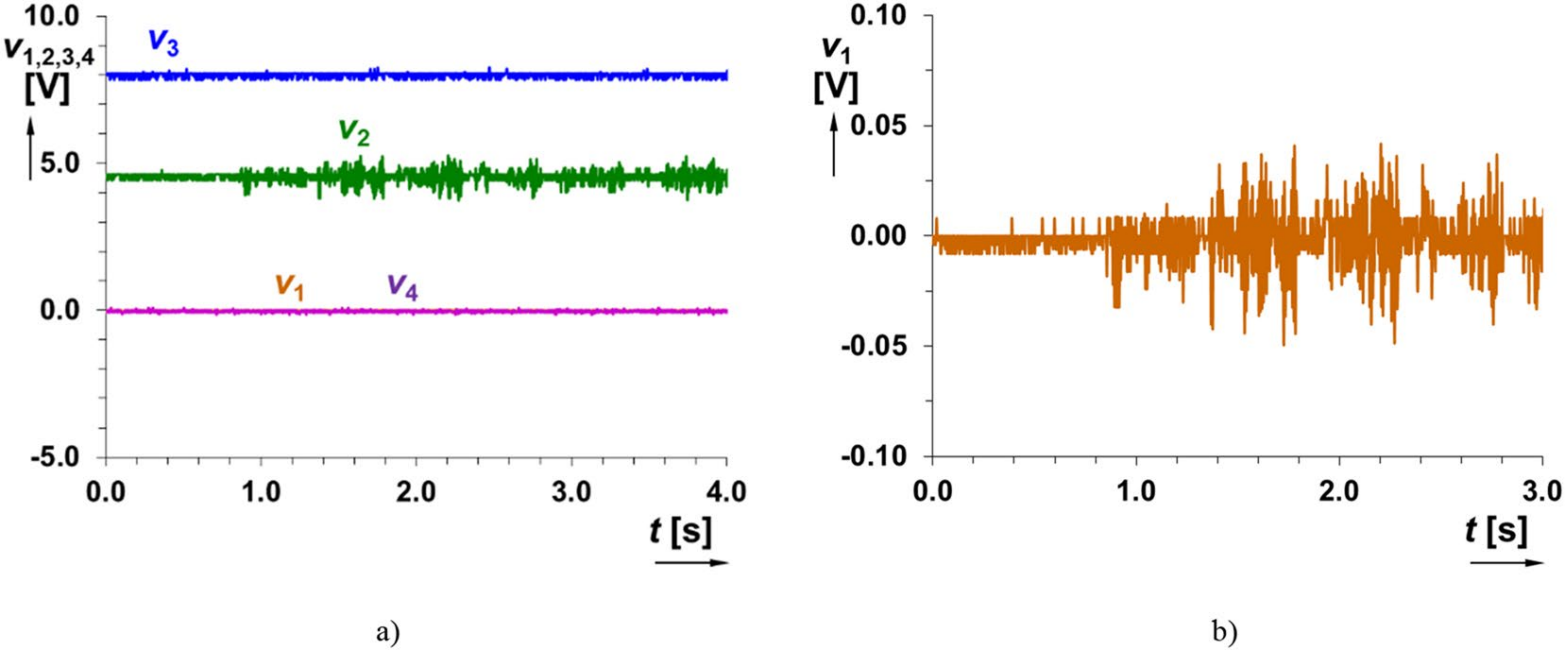
Time domain response of the readout system on talking (voice signal with low level): (**a**) all indicated intermediate and output voltages of the system, (**b**) detail on the signal at the output of microphone (*v*_1_(*t*)).

The signal levels (standardly loud voice of one vowel sound in distance up to 1 m from device) created by talking, depend on the distance between the source of the signal and microphone, but these effects (noises, music, talking, etc.) are unable to initialize the output impulse in the tested cases. The experimental results are plotted in Fig. 6. Signals *v*_1_(*t*) and *v*_4_(*t*) have almost zero value. The signal from microphone has a DC offset around 3.15 V (before AC coupling) in the tested case, but the figures show this signal without offset for better visibility in large voltage scale of vertical axis after AC coupling (*C*_V1_, *v*_1_(t)). The maximum amplitude of the measured signal from the microphone is up to 50 mV (see Fig. 6b). It is visible that the device is designed to not react on this level of voice signal (signal is not detected at the output of the chain). Thereby, the output impulse is not generated, as required.

### Hand-clapping

**Figure 7:**
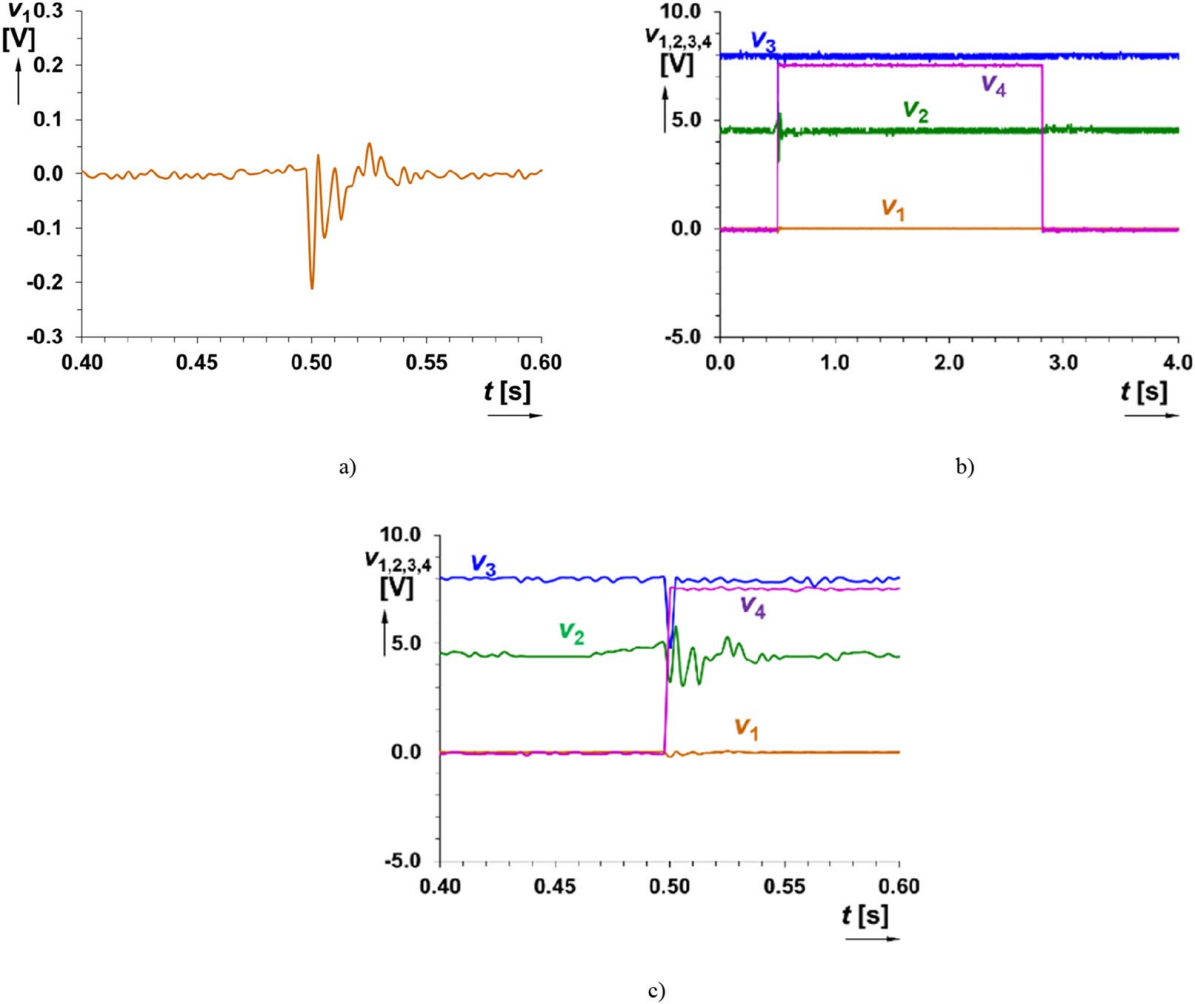
Time domain response of the readout system on handclap: (**a**) detail on the signal at the output of microphone (*v*_1_(*t*)), (**b**) all indicated outputs of the system and (**c**) detail on initialization.

In the second tested use case (single hand clap in approximately distance 0.5 m), the microphone generates “burst” that can reach even low hundreds of mV in amplitude. Time domain response of the readout system on hand-clapping is shown in Fig. 7. The comparator (trigger) reacts by generation of a single drop of value *v*_3_ from level H (high) to level L (low) and return back to H. It initializes the start of waveform *v*_4_(*t*) with duration of 2.32 s. Therefore, the correct setting of sensitivity (i.e. detectable level) fulfills requirement on hand-clap initialization of trigger as expected but is insensitive to voice signal. The voltage level (peak value) sufficient for initialization was set approximately as 200 mV (the threshold of sensitivity for sensed signal produced by a microphone).

### Direct hit on the microphone

The last tested use case (direct hit on the microphone) represents over-excitation of the system. More precisely, it leads to the limitation of the voltages (nonlinear effects) in linear part of the system (amplifier), but it does not have influence on the correct operation of the system. The long limited bursts generated by the microphone (> 500 mV in amplitude) present the most important influence. However, such a behavior is not undesirable because the device still correctly reveals the event. It explains the reason of utilization of the monostable flip-flop in the system. This part of the signal processing chain prevents unwanted generation of several impulses due to immunity of the flip-flop against triggering through the duration of temporarily stable state and recovery time^18^. Thereby, the feature of this part is advantageous for correct behavior of the system. It eliminates nonlinear effects like multiple triggering from a single event.

**Figure 8:**
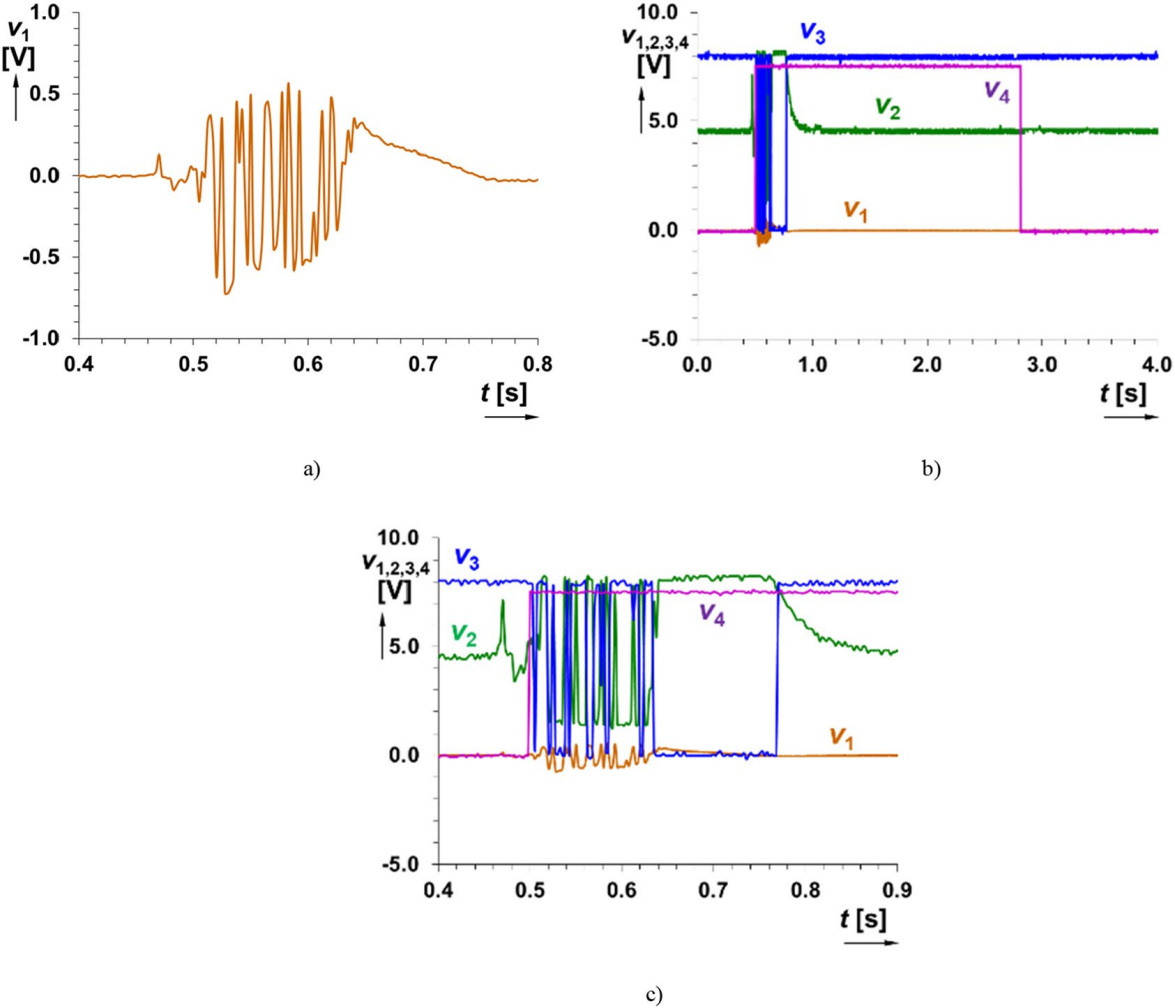
Time domain response of the readout system on direct hit on microphone (signal intentionally limited and level overridden): (**a**) detail on the signal at the output of microphone (*v*_1_(*t*)), (**b**) all indicated outputs of the system and (**c**) detail on initialization.

**Figure 9:**
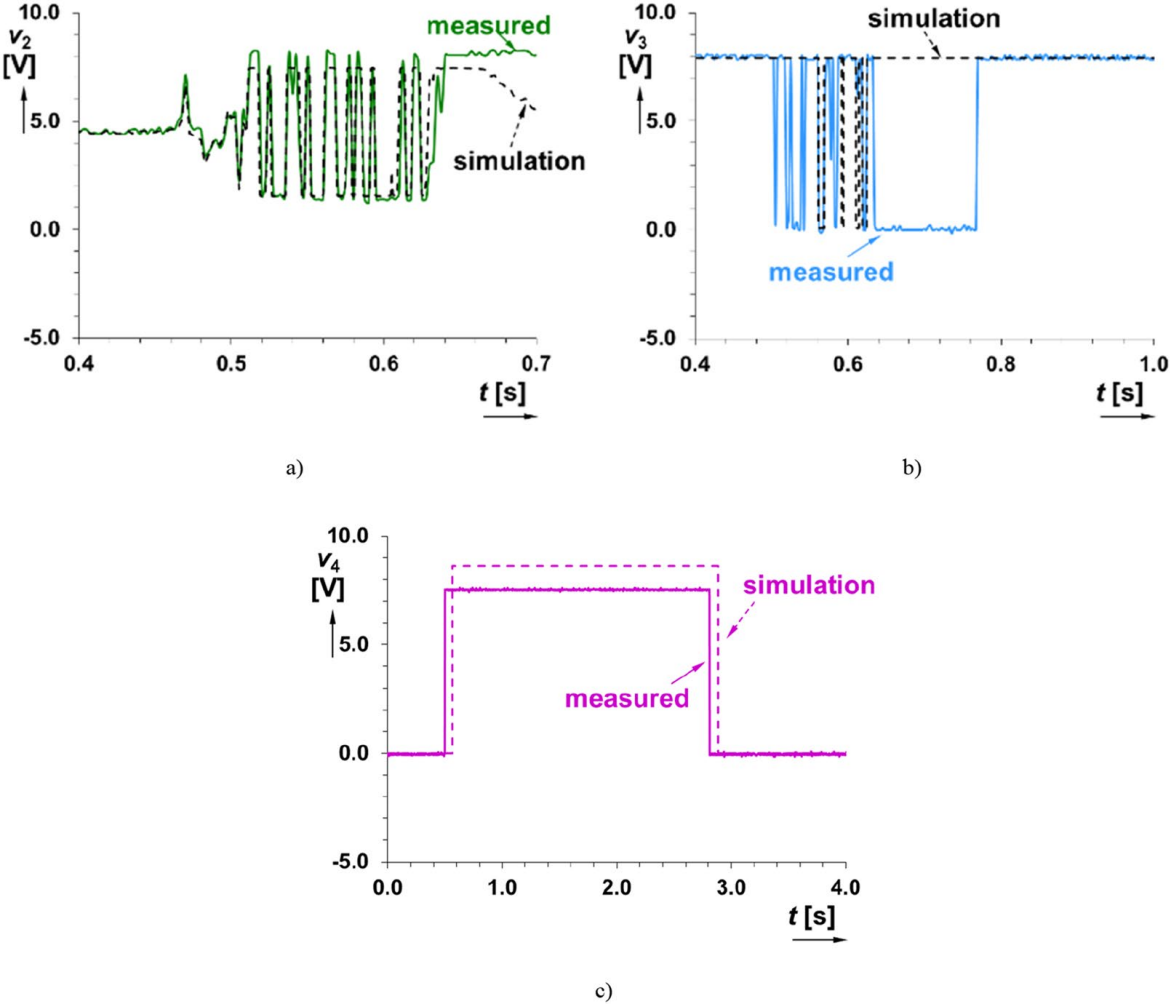
Comparison of simulated and measured time domain response of the readout system on direct hit on microphone (intentionally limited and overridden): (**a**) *v*_2_(*t*), (**b**) *v*_3_(*t*) and (**c**) *v*_4_(*t*).

Triggering and output wave generation is independent on the detailed shape of the processed signal. The initialization of the system depends on the presence of signal above some level set in the comparator part (as well as overdriving in signal paths is allowed and expected, the signal path consists of protecting parts—diode limiters). The described operation is shown in Figs. 8 and 9.

Comparison of significant results with simulations (possible also in previous cases but omitted for better clarity of figures) is shown in Fig. 9. As it is visible, outputs of simulation (the same data as in experiments recorded by microphone and exported to PSpice) well correlated with the outputs of measurements. However, some details of waveforms can be slightly different due to different behavior of real circuit elements. Nevertheless, the initialization of *v*_4_(*t*) is ensured identically in both simulations and measurements.

The quiescent power consumption of the whole device is around 90 mW. When the output impulse is generated (including LED indication), it is around 216 mW. It means life duration of 42 h for maximal power consumption (24 mA) and 100 h for quiescent power consumption (10 mA). These estimations considered the standard 9 V 6F22/PP3 Lithium battery (0.8–1.2 Ah capacity; estimation done for 1 Ah). It must be noted that these values are not high because the device is not under operation frequently (majority of the consumption consists in quiescent operation) and also the device is not under operation permanently (powered all the time). The races (non-professional fire fighters - competition of teams of local fire departments) have duration of several hours approximately. Hence, power requirements are insignificant.

## Processing of gunfire signature

In this section, we present a simulation-based performance study of our proposed concept processing acoustic wave generated by gunfire. Real experiments have not been permitted in our research facilities. For this purpose, we used a signal pattern with signature of a gunshot, available on website^28^, and extracted data points in order to simulate source of this acoustic impact incoming to the input of the readout in PSpice simulator.

**Figure 10:**
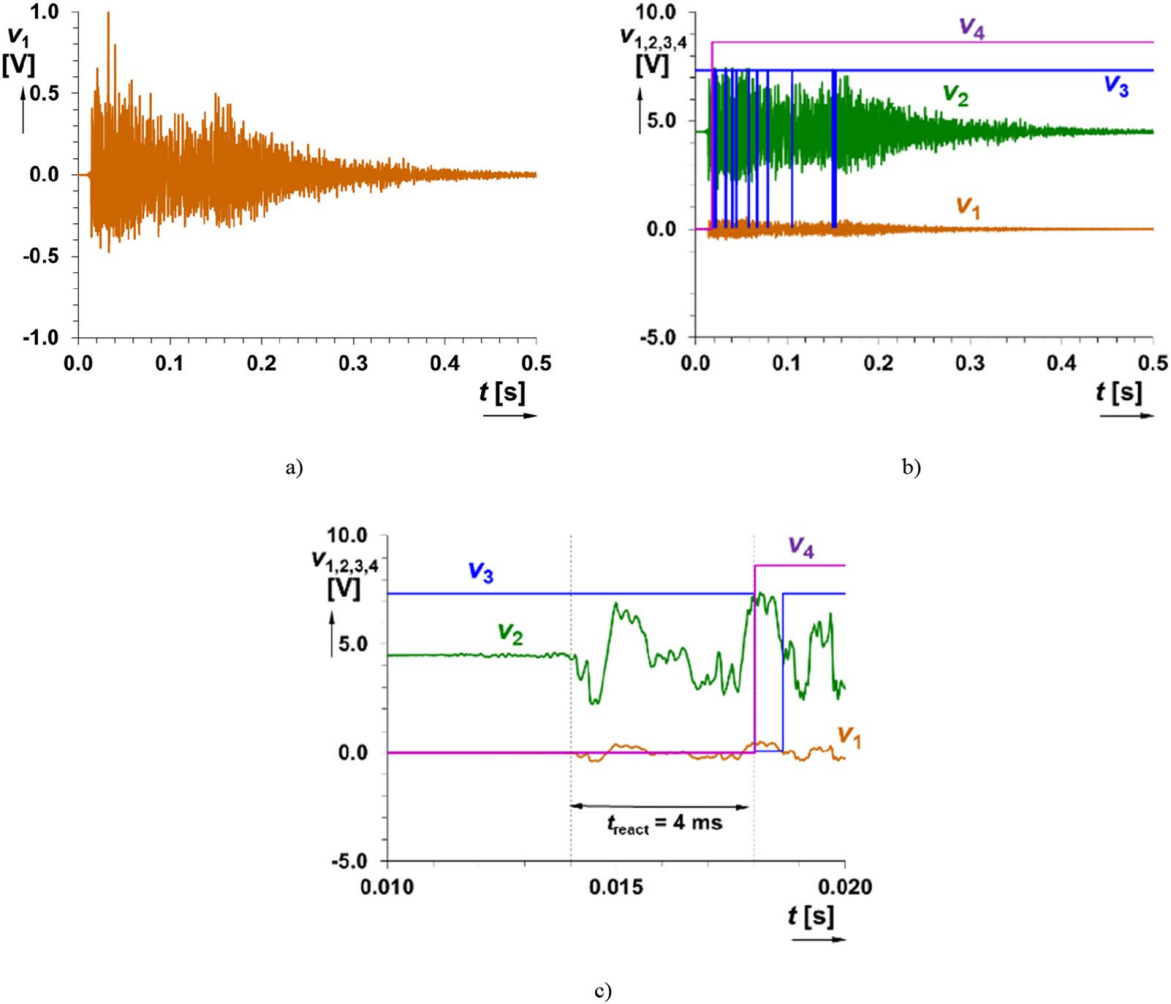
Simulation of time domain response of the readout system on signal pattern of a gunshot (threshold of reaction set to 0.33 V): (**a**) detail on the signal at the input of the system (*v*_1_(*t*)), (**b**) all indicated outputs of the system and (**c**) detail on initialization.

**Figure 11:**
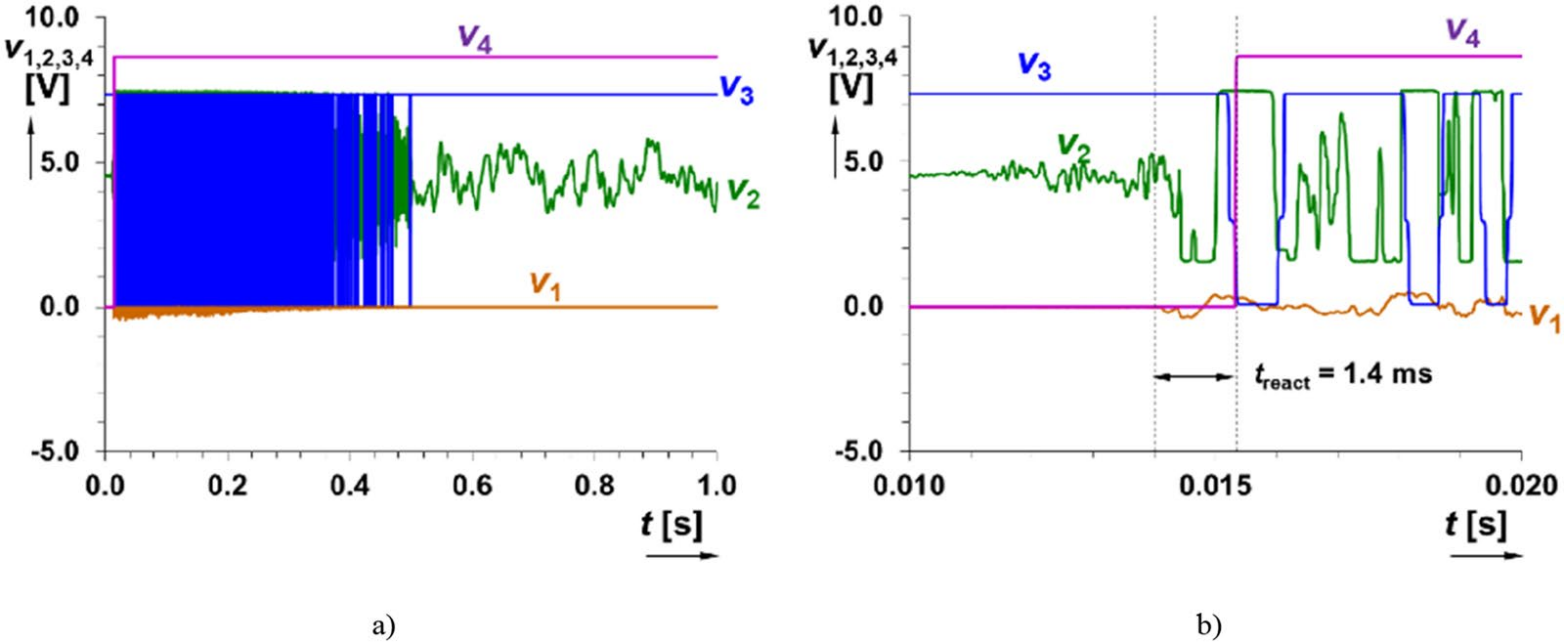
Simulation of time domain response of the readout system on signal pattern of a gunshot (signal intentionally limited and level overridden for threshold of reaction 50 mV): (**a**) all indicated outputs of the system and (**b**) detail on initialization.

Current setting of threshold voltage (*V*_IN_HL_) and gain of the amplifier defines approximate sensitivity (threshold of reaction) of the system. Optimal readjustment of threshold voltages of the comparator block (*V*_IN_HL_ = 2 V, *V*_IN_LH_ = 1 V, *R*_c1_ = *R*_c3_ = 15 kΩ and *R*_c2_ = 2.5 kΩ) offers narrower hysteresis window (Δ*h* = 1 V) that is still sufficient for this application. The sensitivity of the system was set for input threshold amplitude reaction larger than 0.33 V (used lower gain of the amplifier). The obtained results are shown in Figs. 10 and 11. All figures show a detail of time window limited to 0.51 s, but trigger uses setting for *t*_i_ = 2.3 s. The generation of the initializing impulse is not affected even for large amplification and limitation (flip-flop part creates protection against any further changes in the sensed signal), see Fig. 11 (threshold of reaction 50 mV). Therefore, highly accurate setting of the system is not necessary. However, as expected, it influences reacting time (*t*_reac_) of the initialization on input acoustic event (in units of ms). Note that voice and similar signals have several times lower amplitudes (low tens of mV produced by microphone in real case).

## Conclusion

In this paper, a simple readout for precise acoustic event initialization in sport race was introduced. The presented simulation and experimental results verified the performance of the realized readout circuit to detect an acoustic wave generated by various sources (intended for a hand clapping or gunshot for example).

The readout circuit indicates this event behaving in range of hundreds of Hz (−3 dB limit of the filter at 1.6 kHz) by generation of a stable output impulse with settable duration, easily compatible with TTL (5 V) or CMOS (3.3 V) digital logic levels when suitable pull-up resistor to the corresponding voltage level is added. The internal amplifier ensures the amplification of the useful low-frequency signals (fitting spectral character of the expected signals) from the microphone approximately from 6 up to 40 dB with bandwidth about 1.6 kHz in all cases. The device is able to generate a stable impulse (with duration from hundreds of μs up to 2.3 s) for the signal from microphone having certain duration and causing multiple switching of the comparator even when using large hysteresis window. The operationability of the device was tested at the distance of 4 m (the results of tested cases are shown for 0.5 m). All these features and possibility of simple adjustment of sensitivity allow clear evaluation of use cases with low-level surrounding signals (e.g. talking, music, noises, other disturbances) and its simple interpretation for computer processing (synchronized start of stopwatch). The outputs of experimentally tested uses cases confirmed usefulness of the proposed readout for sport races, for instance. The basic concept of microphones and amplifiers with filters in processing chain, as shown in^12^, is insufficient for precise initialization (generation) of single impulse indicating acoustic wave impact. It is due to presence of bursts and overshoots in sensed waveform causing false initialization if not solved by further processing as shown in our work. The proposed analog readout has useful features among similarly simple and low-cost digital solutions (microcontroller units) using only microphone, amplification and antialiasing filtering before digitalization and all further steps solved in fully digital form because of real time analog evaluation without any additional delay required for standard microcontrollers and software timing. Reacting times are taking units of milliseconds.

We tested the system for several scenarios. Operationability of the device for condition of gunfire initialization depends on the threshold voltage based on current sensitivity that can be modified in comparison with handclapping as documented by simulations with sample of gunshot time-domain signature.

## Data Availability

Not applicable.
